# Tolerability and Safety of a Nutritional Supplement with Potential as Adjuvant in Colorectal Cancer Therapy: A Randomized Trial in Healthy Volunteers

**DOI:** 10.3390/nu11092001

**Published:** 2019-08-24

**Authors:** Marta Gómez de Cedrón, José Moises Laparra, Viviana Loria-Kohen, Susana Molina, Juan Moreno-Rubio, Juan Jose Montoya, Carlos Torres, Enrique Casado, Guillermo Reglero, Ana Ramírez de Molina

**Affiliations:** 1 Molecular Oncology and Nutritional Genomics of Cancer, IMDEA-Food Institute, CEI UAM+CSIC, Madrid 28049, Spain; 2 Molecular Immuno-Nutrition Group, IMDEA-Food Institute, CEI UAM+CSIC, Madrid 28049, Spain; 3 Nutrition and Clinical Trials Unit, GENYAL Platform IMDEA-Food Institute, CEI UAM + CSIC, Madrid 28049, Spain; 4 Precision Oncology Laboratory (POL), Infanta Sofía University Hospital, Madrid 28702, Spain; 5 Department of Radiology, Rehabilitation & Physiotherapy, Faculty of Medicine, Complutense University, 28040 Madrid, Spain; 6 Production and Characterization of Novel Foods, CIAL, CEI UAM+CSIC, Madrid 28049, Spain; 7 Production and Development of Foods for Health, IMDEA-Food Institute, CEI UAM+CSIC, Madrid 28049, Spain

**Keywords:** *Rosmarinus officinalis L*., alkylglycerols, immuno-nutrition, colon cancer

## Abstract

Bioactive supplements display relevant therapeutic properties when properly applied according to validated molecular effects. Our previous research efforts established the basis to develop a dietary supplement based on a *Rosmarinus officinalis* supercritical extract. This was enriched in phenolic diterpenes (RE) with proven properties against signaling pathways involved in colon tumorigenesis, and shark liver oil rich in alkylglycerols (AKG) as a bioactive lipid vehicle to improve RE bioavailability and synergize with the potential therapeutic action of the extract. Herein, we have investigated the tolerability and safety of the supplement and the biological and molecular effects from an immuno-nutritional perspective. Sixty healthy volunteers participated in a six week, double-blind, randomized parallel pilot study with two study arms: RE-AKG capsules (CR) and control capsules (CC). Mean age (±SD) of volunteers was 28.32 (±11.39) and 27.5 (±9.04) for the control and the study groups, respectively. Safety of the CR product consumption was confirmed by analyzing liver profile, vital constants, and oxidation markers (LDLox in blood and isoprostanes and thromboxanes in urine). The following were monitored: (1) the phenotyping of plasmatic leukocytes and the ex vivo response of lipopolysaccharide (LPS)-stimulated peripheral blood mononuclear cells (PBMCs); (2) expression of genes associated with immune-modulation, inflammation, oxidative stress, lipid metabolism, and tumorigenesis; and (3) the correlation of selected genetic variants (SNPs) with the differential responses among individuals. The lack of adverse effects on liver profile and oxidation markers, together with adequate tolerability and safe immunological adaptations, provide high-quality information for the potential use of CR as co-adjuvant of therapeutic strategies against colorectal cancer.

## 1. Introduction

Nowadays, there is an increasing interest in nutritional interventions because of their potential role in preventing colon tumorigenesis. Colorectal cancer is the third most prevalent cancer type in males and the second in females worldwide, displaying an increasing incidence even in traditionally low-risk countries such as Spain. In the last years, intervention strategies boosting the activity of the immune system are preferable, together with vehiculation of bioactive components with proven anticancer efficacy. Promotion of proper intestinal mucosal barrier function, cellular defense, and local or systemic inflammation are the main targets of these interventions [1]. Impairment of the regulatory processes of these aspects represent a potential risk of enhanced growth and metastatic ability of cancer cells due to immune dysfunction [2,3]. In the clinical setting, formulas based on different nutrients (i.e., arginine, glutamine, nucleotides, and/or ω-3 fatty acids) have demonstrated benefits in surgical patients by mean of the reduction of inflammatory responses and/or infections [4,5,6]. These beneficial effects have been recognized by ESPEN (European Society for Clinical Nutrition and Metabolism) [7] and ASPEN (American Society of Parenteral and Enteral Nutrition) [8]. A meta-analysis has also demonstrated their effectiveness in diminishing inflammation and enhancing host immunity, although benefits in the clinical outcome were inconsistent [9].

In this scenario, nutritional bioactive co-adjuvants can also help by influencing innate immune signals, which stem at the intestinal level to drive an adequate maturation of adaptive immunity. Nutritional interventions, in combination with classic pharmacological cancer treatments, represent a promising strategy to increase the number of patients which could respond successfully against immune–metabolic-based diseases derived from chemotherapy. The clinical seek for agents with favorable tolerability and safety profiles which could be incorporated as co-adjuvant players to pharmacological anticancer treatments makes also attractive the bioactive supplements. In cancer patients, activation of type-1 innate and acquired immunities are crucial for tumor immunotherapy to overcome immunosuppression [10]. Immune response(s) depends on a multitude of factors such as the offending agent, the microenvironment of the effector cells, and the host’s capacity to respond. Moreover, it must constantly be adaptive and be able to integrate responses from many different cell types. The first line of defense of the innate immune system is the gut mucosa, which not only acts as the primary barrier, but it can also drive adequate adaptive immune responses by influencing its maturation processes. In this sense, dendritic cells (DCs), monocytes (MC), macrophages (Mθ), and other antigen-presenting cells (APCs) are upstream of T helper (Th) responses. Thus, understanding how naturally-occurring preventive/therapeutic agents selectively influence tolerance and immune cell function can set the stage for a new generation of active cancer immunotherapies. The latter could represent a path forward to develop durable, long-lasting immune responses against cancer [11].

Herein, we have investigated the immune-nutritional tolerability and safety of a supplement based on a *Rosmarinus officinalis* supercritical fluid extract enriched in phenolic diterpenes (RE) and shark liver oil enriched in alkylglycerols (AKG) as a bioactive lipid vehicle [12] to be used as a potential co-adjuvant in intervention strategies for specific patients suffering from colorectal cancer and/or immune disorders.

## 2. Study Design and Materials and Methods

### 2.1. Supplement Composition

As we have previously demonstrated, specific ranges of carnosic acid/carnosol composition of supercritical fluid *Rosmarinus officinalis L* extracts (RE) display relevant antitumor effects in colon and breast cancers [13]. Moreover, RE synergizes with 5-Fu and sensitizes 5-Fu-resistant colon cancer cells to this drug [14]. However, due to the low bioavailability of the bioactive compounds present in plants [15], and with the aim to obtain therapeutic benefits of these compounds in the clinical setting, we have developed a supplement of RE, approved for human use, together with a vehicle system based on bioactive alkylglycerols (AKGs), also approved for human use (PCT/ES2017/070263). The current strategy was developed to enhance bioavailability of RE and to potentiate the antitumor effects of RE with bioactive alkylglycerols. For this, 60 healthy volunteers were randomized to participate in the study: 30 received the study soft gelatin capsules (CR)—rosemary supercritical extract (11.25 mg of diterpene phenols) and shark liver oil (SLO) enriched in AKGs (150 mg)—and another 30 volunteers received the control capsules (CC). Detailed composition of the study (CR) and control capsules (CC) are indicated in Supplementary Table S1. The study and control capsules had the same coating and appearance, and capsules were consumed once a day for six weeks. The randomization was performed blindly. The company that produced the product was the only one that knew which group corresponded to each product

### 2.2. Subjects and Study Design

The set-up of the clinical trial consisted of a six week, double-blind, randomized and parallel pilot study with two study arms—RE and alkylglycerol containing capsules (CR) and control capsules (CC)—to evaluate the immunomodulatory effect (by quantifying changes in peripheral blood leukocyte subpopulations and the cytokine profile produced by peripheral blood mononuclear cells (PBMCs) after the ex vivo lipopolysaccharide (LPS) stimulation) (primary outcome), together with the modification of oxidation markers and modulation of the expression of genes related to immune responses, inflammation, oxidative stress, lipid metabolism, and cancer in healthy volunteers. Moreover, association of selected genetic variants (SNPs) with the responses to the intervention were also analyzed (secondary outcomes).

The pilot study protocol was approved by the local Ethics Committee of the IMDEA Food Foundation (IMD PI017), and it was carried out in accordance with The Code of Ethics of The World Medical Association (Declaration of Helsinki). Written informed consent was obtained by all subjects prior to starting the trial. Clinical Trial Registry number: This trial was registered at clinical trials.gov as NCT03492086 http://clinicaltrials.gov/. PATENT: (PCT/ES2017/070263). 

Volunteers were recruited from the Campus of Cantoblanco and Parque Científico de Madrid, from the GENYAL Platform, and from the dissemination of the study through the IMDEA Food Foundation web page and other means of communication.

Of the 123 volunteers interested in participating in the study, 63 were excluded before randomization for not meeting the inclusion–exclusion criteria of the study, not attending the screening visits (V0), or because they decided not to participate due to distance or personal reasons. Inclusion criteria: Age between 18 and 55 years; adequate understanding of the study; and willingness to complete the entire treatment. Exclusion criteria: Body mass index (BMI) >30 kg/m^2^; diagnosis of diabetes mellitus (T2D), hypertension, dyslipidemia, or other cardiometabolic disorders; impaired cognitive function; diagnosed hepatic, renal, or cardiovascular disease; subjects with primary immunodeficiency disorders, consumption of drugs with influence on the immune system, or splenectomy; presence of other pathologies like asthma, food allergies, Crohn’s, myasthenia gravis, or lupus; consumption of vitamins, minerals, supplements of antioxidant extracts, or protein supplements in the two weeks prior to the start of the study; subjects treated with drugs affecting the lipid or glycemic profile (i.e., statins, fibrates, diuretics, corticoids, anti-inflammatories, hypoglycemic agents, or insulin) during the previous 30 days; consumption of anticoagulants or antiplatelet agents, cyclosporine, acetylsalicylic acid, antihistamines, or sedatives; hypersensitivity to rosemary, to its components or other members of the family of lipped plants, or to soybean as excipient of the capsules; allergy or hypersensitivity to fish; habitual smoking or high consumption of alcohol; pregnant or lactating women; and high-intensity physical exercise.

Due to the lack of previous studies carried out with the products under study, a pilot study was carried out with a sample size of 60 volunteers (control group–CC: 18 females and 12 males; and study group–CR: 18 females and 12 males). From this study, we intended to calculate the sample size of subsequent studies, taking into account the main efficacy variable—with a final 60 subjects. The randomization procedure was provided by the Biostatistics Unit of the IMDEA Food Foundation. It was carried out using a randomization table generated by Statistic Software R 2.15 version (www.r-project.org, University of Auckland, Auckland, New Zealand.)

The randomization and masking data were strictly confidential. Once the study was completed, the data file was verified and, after performing the results analysis, the codes were opened to proceed with the interpretation of the same. All volunteers registered a complete nutritional survey: 3-day habitual food registry and questionnaires about physical activity, capsule tolerance, and capsule consumption.

Before the first study visit, a group CC volunteer decided to retire due to having problems to attend to, and another volunteer had to be withdrawn due to increased levels of liver enzymes in the baseline analytical group CC, so that the study was finally initiated by 58 volunteers (35 females and 23 males): 28 in group CC (17 females and 11 males) and 30 in group CR (18 females and 12 males). Throughout the study, one volunteer was withdrawn as a precaution since she had persistent headaches, which turned out to be in group CC, so 57 participants finished (27 in group CC and 30 in group CR). No exclusion was made prior to the analysis, since all volunteers registered a consumption ≥75% of the agreement.

Blood samples were drawn and anthropometric data, bioelectric impedance analysis (BIA), and vital constants were monitored at each visit: V1 (baseline parameters), V2 (control visit), and V3 (final parameters). Morning urine was also collected (V1, V3).

**Figure 1:**
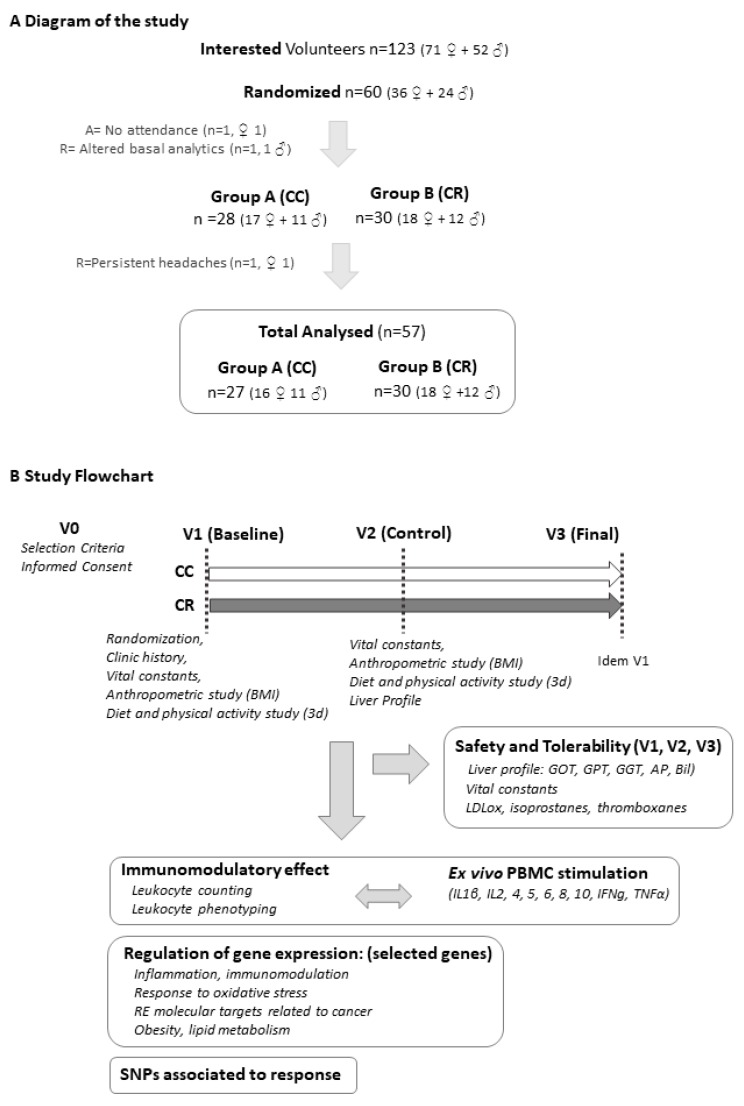
(**A**) Diagram of the study. (**B**) Study flowchart, objectives, and analyzed parameters. Abbreviations: CC, control capsules; CR, intervention capsules; BMI, body mass index; PBMC, peripheral blood mononuclear cell; SNPs, single nucleotide polymorphisms.

Primary outcome: The immunomodulatory effect of the intervention was assessed by analyzing: (i) The levels of cytokines (IL-1β, IL-2, -4, -5, -10, IFNγ, TNFα) produced by PBMCs after ex vivo LPS stimulation; (ii) the effect on the hematological profile and differential counts of leukocytes; and (iii) the phenotyping of leukocyte subpopulations. Secondary outcomes: (iv) Modification of parameters related to inflammation and oxidative stress; and (v) modulation of the expression of genes associated with immune and inflammatory responses, lipid profile, oxidative stress markers, and cancer (from a panel of 48 specifically-selected genes). Single nucleotide polymorphism (SNP) analysis was included to identify putative associations with the differential responses to the intervention. Figure 1A shows a diagram of the study and Figure 1B displays the study flowchart and main objectives.

### 2.3. Biochemical Analyses

Triacylglycerol (TG), total cholesterol (TC), low-density (LDL-C) and high-density (HDL-C) cholesterol concentrations were measured by routine laboratory (Laboratorio CQS, Madrid, Spain, which follows the UNE-ISO 15189:2007 directives) methods. Urea, creatinine, hepatic enzymes—glutamyl oxaloacetic transaminase (GOT), glutamic-pyruvatetransaminase (GPT), and gamma glutamyltransferase (GGT)—bilirrubin, and alkaline phosphatase (AP) were also determined. The concentration of LDLox was measured by sandwich enzyme-linked immunosorbent assay (ELISA) by using the monoclonal antibody mAb-4E6 (Mercodia AB, Uppsala, Sweden). Urinary thromboxane B2 (TBX2) and total isoprostanes were quantified by competitive ELISA (Enzo Biochem, Inc., New York, USA and Oxford Biomedical Research, Michigan, USA, respectively).

### 2.4. Isolation of Peripheral Blood Mononuclear Cells (PBMCs)

Subjects were instructed to fast overnight before each blood collection. Blood samples were collected in heparinized tubes (BD Vacutainer, Franklin Lakes, NJ, USA) at each visit between 08:00 and 10:00 to minimize circadian variations, which processed within 2 h of collection and used for PBMCs isolation. Isolation was carried out under sterile conditions to avoid monocytes activation. Briefly, whole blood was diluted (1:1) with phosphate buffer solution (PBS) and centrifuged by density gradient with Histopaque-1077 (Sigma–Aldrich, Madrid, Spain), according to the manufacturer’s instructions. After collection, PBMCs were washed twice with PBS.

### 2.5. Multiplex Bead Immunoassay

For the analysis of the ex vivo cytokine profile produced by PBMCs after LPS stimulation, isolated PBMC were first incubated for 12 h and then treated with LPS. Supernatants were recovered to determine concentrations of IL-1β, IL-2, IL-4, IL-5, IL-6, IL-8, IL-10, IFNγ, and TNFα using a magnetic bead-based immunoassay (Human High Sensitivity T Cell Magnetic Bead Panel A MAGPIX-Luminex) kit from Millipore Iberica SA (Madrid, 28050, Spain), following the manufacturer’s instructions. A minimum of 50 beads per parameter were analyzed by the MAGPIX-Luminex system (Millipore Iberica SA (Madrid, 28050, Spain). Raw data (median fluorescence intensity, MFI) were analyzed with the xPONENT software 4.1 (Luminex system, Millipore Iberica SA (Madrid, 28050, Spain).

### 2.6. FACS (Fluorescence Activated Cell Sorting) Analysis of Cluster of Differentiation (CDs) Markers

Analysis of cluster differentiation markers was achieved by flow cytometry with a Gallios analytical flow cytometer (Beckman Coulter, Irving, TX, USA). Aliquots (100 µL) of peripheral blood were mixed with the following fluorochrome-conjugated antibodies: Anti-human CD3-APC, CD4-APCeF750, CD8-PC7, CD14-PC5.5, CD16-PB, CD45-KrO, CD54-FITC, and CD56-PE (Beckman Coulter, Irving, TX, USA), and lysed with the Beckman Coulter Versalyse solution following the manufacturer’s recommendations. To obtain the cell counts for the different populations 100 µL of Flow-Count Fluorospheres (Beckman Coulter, Irving, TX, USA) were added.

### 2.7. Cell Culture and In Vitro Treatments

Human colon cancer cells SW620 were obtained from American Type Culture Collection (ATCC, Manassas, VA, USA). SW620 cells were cultured in DMEM supplemented with 10% of fetal bovine serum (FBS) and 2 mmol/L glutamine. Cells were maintained under standard conditions of temperature (37 °C), humidity (95%), and carbon dioxide (5%).

For the in vitro validation of the molecular targets found in the clinical trial, we treated SW-620 CRC cells with three different doses corresponding to 0.5 × IC_50_, 1 × IC_50_ and 2 × IC_50_ for 4, being the IC_50_ value in this cell line of 36.46 ± 7.23 mg/mL as previously described [14].

### 2.8. RNA Extraction

Total RNA was extracted and purified from homogenized PBMCs with miRNeasy minikit, (Qiagen, Valencia, CA, USA) following the manufacturer’s protocol. Recovered RNA’s concentration and integrity were verified using a Nanodrop ND-1000 spectrophotometer (Nanodrop Technology, Cambridge, UK).

### 2.9. Gene Expression Analysis

After DNAse I treatment (Invitrogen, Madrid, Spain), 1 g of total RNA was reverse transcribed for 2 h at 37 °C with miScript^®^ II Reverse Transcription kit (Qiagen, Germantown, MD, USA), according to the manufacturer’s guidelines. A Taq-Man Low Density Array (Applied Biosystems, Madrid, Spain) was specifically designed for this experiment, including 47 selected genes (Supplementary Table S2) linked to immune system, inflammation, oxidative stress, lipid metabolism, and cancer-associated genes. Gene-expression assays were performed in a HT–7900 Fast Real-Time PCR. GAPDH was used as endogenous control. RT-StatMiner software (Integromics^®^ Inc., Madison, WI, USA) was used to detect and determine the quality control and differential expression analyses. The Expression Suite Software (Life Technologies, Madrid, Spain) program was used to obtain the Ct data. The ΔCt (Ct gene-Ct GAPDH) was calculated, and then the relative expression (RQ) between visits was calculated (V3−V1) following the 2^−ΔΔCt^ method [16].

### 2.10. DNA Extraction and Genotyping

Genomic DNA from each participant was isolated from 300 μL of total blood using the QIAamp DNA Blood Mini Kit (Qiagen Sciences, Inc, Germantown, MD, USA) and recovered in 100 μL of nuclease-free water. Concentration and quality were then measured in a nanodrop ND-2000 spectrophotometer (ThermoScientific, Waltham, MA, USA). Genotyping was performed using the QuantStudio 12 K Flex Real-Time PCR System (Life Technologies Inc., Carlsbad, CA, USA) with a TaqMan OpenArray plates. Sixty-four single nucleotide polymorphisms (SNPs) were selected on the basis of their known involvement in different parts of the pathogenic processes of inflammation, immune system, obesity, lipid metabolism, redox homeostasis, and cancer (Supplementary Table S3) and its related phenotypes. The results were analyzed using the TaqMan Genotyper software (Life Technologies Inc., Carlsbad, CA, USA).

### 2.11. Statistical Analyses

Data were analyzed with the statistical program R Statistical Software version 2.15 (www.r-project.org, University of Auckland, Auckland, New Zealand). Description of qualitative data was made by absolute frequencies and percentages and quantitative data by mean and standard deviation (SD), standard error of the mean (SEM), or 95% confidence intervals (CI), depending on distribution of the data. For comparison of quantitative data between the two groups, Student *t*-test (parametric test) or Mann-Whitney U or Wilcoxon test (non-parametric test) was used. Chi-square test or Fisher’s test were used to compare proportions of qualitative data between the two groups. Two-way repeated-measures ANOVA was used to evaluate differences of the effect of time (visits), treatment (group), and interaction time x treatment. The *p*-value was adjusted by sex, age, and BMI. When significant interaction was observed, post-hoc analysis and the Bonferroni correction were applied.

For the statistical analysis of genetic data, *X*^2^ test was used to establish the deviation of the genotypic frequencies in controls compared with those expected under the Hardy–Weinberg equilibrium. Three-way ANOVA was used to evaluate the interaction time x treatment x genotype.

All the statistical tests were considered bilateral and a *p*-value < 0.05 as significant.

## 3. Results

### 3.1. Baseline Variables and Safety and Tolerability of Product Consumption

To discard differences after randomization between the control (CC) and intervention (CR) groups and to establish the variable basal levels in the study (V1), the following were monitored: (i) hemogram—leucocytes, hemoglobin, erythrocytes, platelets; (ii) lipid profile—total cholesterol (TC), high density lipoprotein (HDL), low density lipoprotein (LDL), triglycerides (TG); (iii) oxidation-inflammation markers—C reactive protein (CRP), LDLox, isoprostanes, tromboxanes, IFNγ, IL-10, IL-1β, IL-2, IL-4, IL-5, TNFβ-; (iv) anthropometry—BMI, weight, hip and waist circumferences, total fat mass (TFM), total muscle mass (TMM); (v) vital constants—systolic and diastolic blood pressures (SBP, DBP); and (vi) heart rate (HR). No significant differences were found between groups in V1 in any of the variables analyzed, indicating proper randomization (Table 1).

To monitor safety of the study, the levels of liver enzymes (GOT, GPT, GGT, AP), total bilirubin, and creatinine were evaluated at V1, V2, and V3 visits. No significant differences were found in any of the variables, nor in the evolution of hematological profile and vital constants, according to the interaction ‘time x treatment’ (Table 2).

The evolution of inflammation and oxidation parameters (total isoprostanes-Tisos, thromboxanes, TBX2; C reactive protein, CRP; and low density lipoprotein oxidized, LDLox) remained unaltered, except for the levels of LDLox (Table 3A). The post-hoc analysis indicates that LDLox levels in the control group were reduced between visits (*p* = 0.0289). Besides, a non-significant (*p* = 0.193) trend was observed to increase LDLox levels in the study group.

The evolution of the lipid profile (total cholesterol, CT; low density lipoprotein, LDL; high density lipoprotein, HDL; and triglycerides, TG) revealed differences in the levels of LDL (Table 3B). The post-hoc analysis showed a significant reduction in the levels of total LDL (*p* = 0.001) only in the control group (CC). The study group (CR) showed a reduction tendency without reaching levels of significance (*p* = 0.191).

Evolution of anthropometric parameters and body composition (weight; body mass index, BMI; percentage of fat mass, FM; percentage of muscle mass, MM) was also analyzed. For the percentage of FM, the differences were found at the significance limit (*p* = 0.0523) (Table 3C). The post-hoc analysis indicates that while the control group (CC) significantly reduced the %FM between visits (*p* = 0.0016), significant changes could not be quantified for the %FM (*p* = 0.6983) in the study group (CR). All in all, these results indicate that the observed reduction in LDL and LDLox levels in the control group (CC) seem to be related to the significant reduction in the percentage of fat mass.

During the intervention period, good tolerance to the product was confirmed by registering a questionnaire about the symptoms related to heartburn, diarrhea, nausea, constipation, swollen belly, bad breath, repeated taste, diuretic effect, and presence of severe gastrointestinal symptoms.

There were no observed differences in the study group (CR) during the time of any of the analyzed variables—liver profile: GOT, GPT, GGT, AP, Bil; creatinine; vital constants; oxidation markers: LDLox in blood and isoprostanes and thromboxanes in urine—supporting safety and tolerability of the product.

### 3.2. Immunomodulatory Effect on Leukocyte Subpopulations

Absolute counts in CD markers—CD45, CD3, CD4, CD8, CD14, CD16, CD56—revealed differences according to the interaction “treatment (CC or CR) x time” (Table 4). The post-hoc analysis indicates a significant increase in CD45^+^ counts between visits (*p* = 0.0008) in the study group (CR), meanwhile the control group (CC) had a tendency towards reduction, although not significant (*p* = 0.7859). CD14^+^, CD16^+^, CD56^+^ significantly increased between visits (V1–V3) in the study group (CR) for CD14^+^ (*p* = 0.0001); CD16^+^ (*p* = 0.0209) and CD56^+^ (*p* = 0.0229). Besides, no significant differences were found in the control group (CC) (*p* = 0.9636, *p* = 0.3363, and *p* = 0.6221, respectively). CD3^+^, CD4^+^ and CD8^+^ cell counts significantly increased in the study group (CR) between visits (*p* = 0.0001, *p* = 0.0001, and *p* = 0.0002, respectively). However, differences were found in the control group (CC) (*p* = 0.2292, *p* = 0.1479, and *p* = 0.4516, respectively).

**Figure 2:**
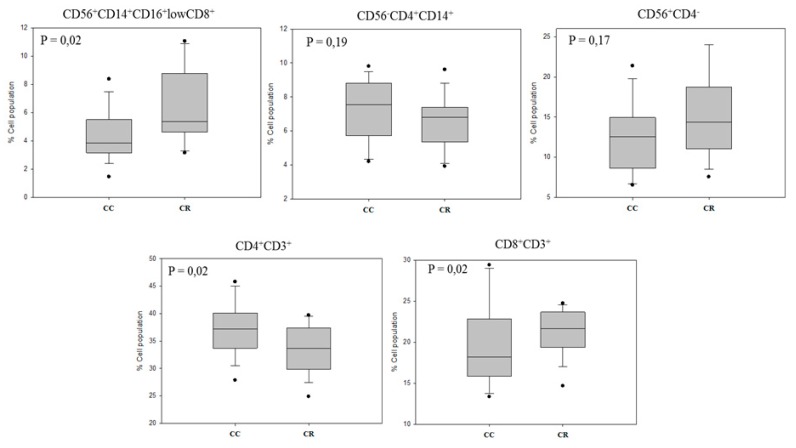
Combined CD analysis to identify leukocyte subpopulations. The phenotyping of leukocyte subpopulations, by combined analysis of CD markers, allows to differentiate among monocytes (CD56^+^ CD14^+^ CD16^+^ lowCD8^+^), macrophages (CD56^−^ CD4^+^ CD14^+^), NK cells (CD56^+^ CD4^−^), T helper (Th) lymphocytes (CD4^+^ CD3^+^), and cytotoxic T lymphocytes (Tcit) (CD8^+^ CD3^+^).

Immunophenotyping of leukocyte subpopulations, by combined analysis of CD markers, allowed us to identify monocytes (CD56^+^ CD14^+^ CD16^+^ lowCD8^+^), monocyte/DC-like (CD56^−^ CD4^+^ CD14^+^), NK cells (CD56^+^ CD4^−^), T helper (Th) lymphocytes (CD4^+^ CD3^+^) and cytotoxic T lymphocytes (Tcit) (CD8^+^ CD3^+^) (Figure 2). Significant changes were found between the treatments: Monocytes (CD56^+^ CD14^+^ CD16^+^ lowCD8^+^) (*p* = 0.02) and cytotoxic T lymphocytes (CD8^+^ CD3^+^) (*p* = 0.02) increased in the CR group, while T helper (CD4^+^ CD3^+^) decreased (*p* = 0.02) compared to the CC group. Overall, these results evidence a polarization of the immune adaptive branch towards a phagocytic and effector cytotoxic response. Also, bearing in mind the key role of CD4^+^ T-helper cells in the generation of CD8^+^ T-cell memory subsets, the cellular response observed supports a non-specific effector response.

### 3.3. Cytokine Profile After Ex Vivo LPS Stimulation of PBMC

**Figure 3:**
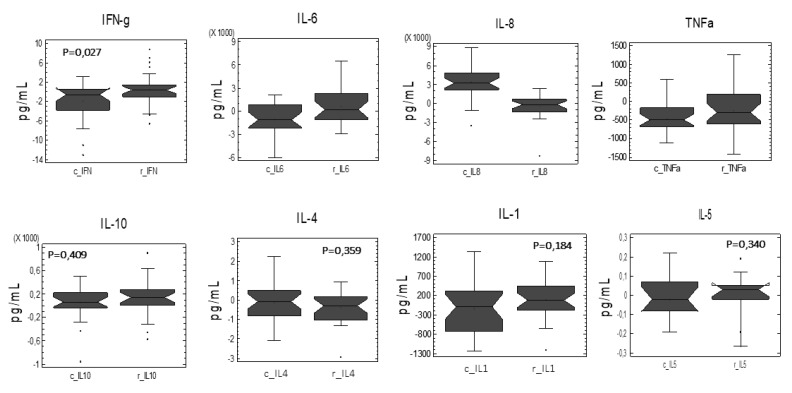
Paired comparisons of the relative variation of each cytokine as a function of the treatment applied. The analysis revealed an increased cellular capacity in response to the LPS stimulation regardless of IFNγ (4-fold, *p* = 0.027) and IL-6 (1.9-fold, *p* = 0.011), while decreased IL-8 (6.6-fold, *p* = 0.001) levels of the CR group compared to control group.

Further studies have tried to evaluate variations in the cellular response capacity to lipopolysaccharide (LPS) from Gram negative bacteria, which potentially occurs from loss of intestinal barrier caused by colon cancer malignancy [17]. For this reason, we compared the cellular response to the ex vivo LPS stimulation of PBMCs from CC and CR groups. Paired comparisons of the relative variation of each cytokine as a function of the treatment applied were performed (Figure 3). The analysis revealed an increased cellular capacity in response to the LPS stimulation regardless IFNγ (4-fold, *p* = 0.027) and IL-6 (1.9-fold, *p* = 0.011), while decreased IL-8 (6.6-fold, *p* = 0.001) levels of the CR group compared to control group. These observations are in accordance with the development of adequate tolerance responses to LPS, which has been previously reported to occur in coordination with the production of IFNγ [18].

To explore the potential relationship of the cytokine profile with the treatment applied, principal components analysis (PCA) plots were also used for the concurrent display of changes in cell cultures subjected to the treatment with CR or CC. The PCA allows to reduce the number of variables and to identify patterns in the data. By expressing the data in such a way, this analysis highlights the similarities and differences of the data according to the treatment (Supplementary Figure S1). PCA analysis, where two components extracted account for 98.3% of the variance, indicates perturbations in the cytokines secreted by PBMCs cultures obtained from patients receiving CR. The two-dimensional chart of PC1 (Eigenvalue, 1.21) and PC2 (Eigenvalue, 0.79) shows the variability of the observations and how the two dimensions change, suggesting the influence of CR on the phenotypic adaptation responses of PBMCs. Collectively, the ex vivo responses to LPS stimulation of the PBMC cultures established from healthy donors that received CR or CC product, suggest that the likely physiologic impact of CR product on immunocompetent cells can be extended successfully to patients and may substantially potentiate immune response(s) in vivo.

The trend in the effector CD4^+^ CD3^+^ cell population associated to the cytokine profile in the individuals administered with CR does not allow us to assume increases in the population of regulatory T cells—Treg—with immunosuppressive character which, generally, in an immature state, are associated with tumors. Moreover, the production of IFNγ favoring a Th1 cellular response usually produces cytotoxic macrophages and effector cytotoxic T cells that would suppose a potential favorable response against tumor development.

### 3.4. Modulation of the Expression of Genes Related to Immunomodulation, Inflammation, Oxidative Stress, and Cancer

Inflammation and immunity coexist at different steps throughout the progression of tumors. Thus, chronic inflammation has been linked to various stages of the tumorigenic process, including proliferation, transformation, invasion, angiogenesis, and/or metastasis. In the tumor microenvironment, the distribution of the immune system subpopulations, together with cytokines and chemokines, dictates the direction of the response towards a protective inflammation or towards tumor promotion. Here, reactive oxygen species (ROS) can also contribute to the development and progression of numerous diseases, including cancer, by modifying DNA, proteins, and/or lipids, and thus increasing the risk of mutagenesis. Moreover, recent studies emphasize obesity as a risk factor in the development of cancer. For these reasons, genes related to inflammation and inflammatory response, immunomodulation, oxidative stress and antioxidant response, fatty acid metabolism, adipogenesis, obesity, and molecular targets of rosemary in cancer are represented in this study. Supplementary Table S2 summarizes the pathways and genes analyzed in this study.

**Figure 4:**
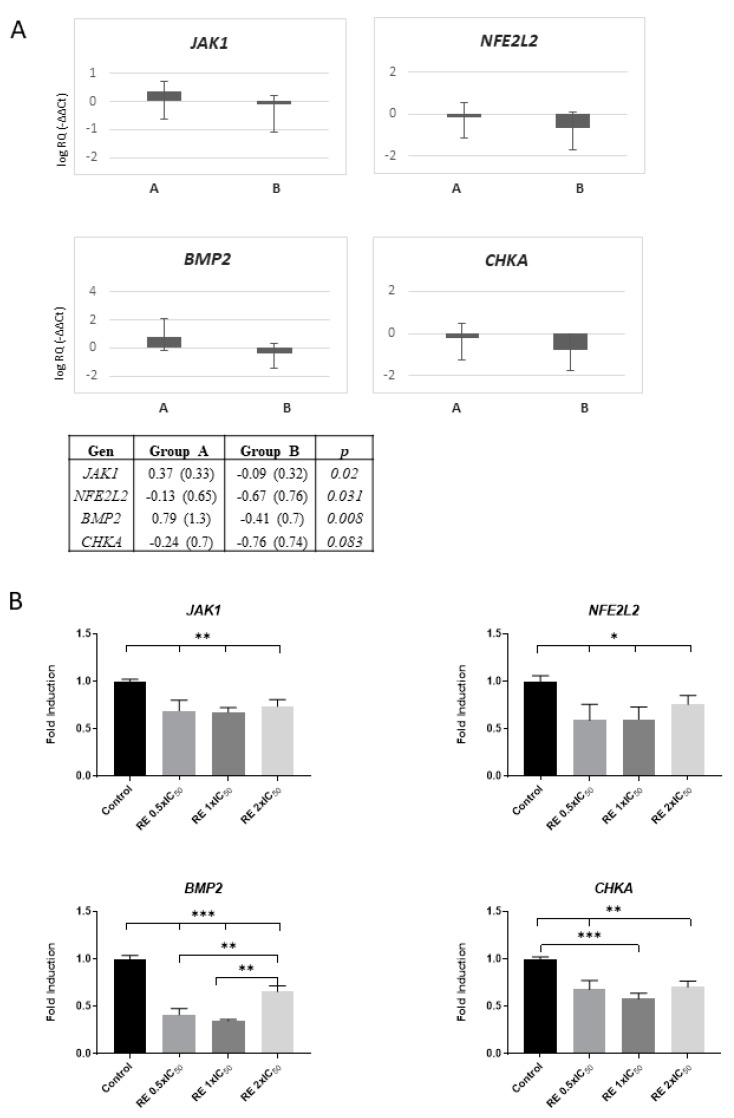
Modulation of the expression of genes related to immunomodulation, inflammation, oxidative stress, and cancer. (**A**) Genes whose expression were statistically different between groups (CC or CR), regardless of evolution between visits, are shown. *JAK1, NFE2L2,* and *BMP2* were significantly decreased in the study group (CR) compared to control (CC) (*p* = 0.02, *p* = 0.031, and *p* = 0.008, respectively). *CHKA* was diminished in the study group (CR) between visits, although not significantly (*p* = 0.083). (**B**) In vitro validation of the effect of rosemary extract in downregulating the expression of *JAK1, NE2L2*, *BMP2,* and *CHKA* in SW-620 colon cancer cells after treatment with three different doses corresponding to 0.5 × IC_50_, 1 × IC_50_, and 2 × IC_50_ for 4, being the IC_50_ value in this cell line of 36.46 ± 7.23 mg/mL, as previously described [14]. *, **, *** = *p*-value < 0.05, 0.01 and 0.005 respectively.

Gene expression analyses show differences in the evolution between groups as a result of the treatment, except *JAK1, NFE2L2, CHKA*, and *BMP2* genes, which significantly diminished in the CR group (Figure 4A). However, the healthy nature of donors could bias these effects. For this reason, we performed in vitro studies in colon cancer cells (SW-620) treated with RE to confirm and validate the specific effect of RE on decreasing the expression levels of *JAK1, NFE2L2, CHKA*, and *BMP2* genes. As shown in Figure 4B, RE diminished the expression levels of these genes, supporting its usefulness as co-adjuvant in colon cancer therapy (further discussed). 

### 3.5. Associations Between Different Genetic Variants (Single Nucleotide Polymorphisms, SNPs) and Responses to the Dietary Intervention with the Capsules

**Figure 5:**
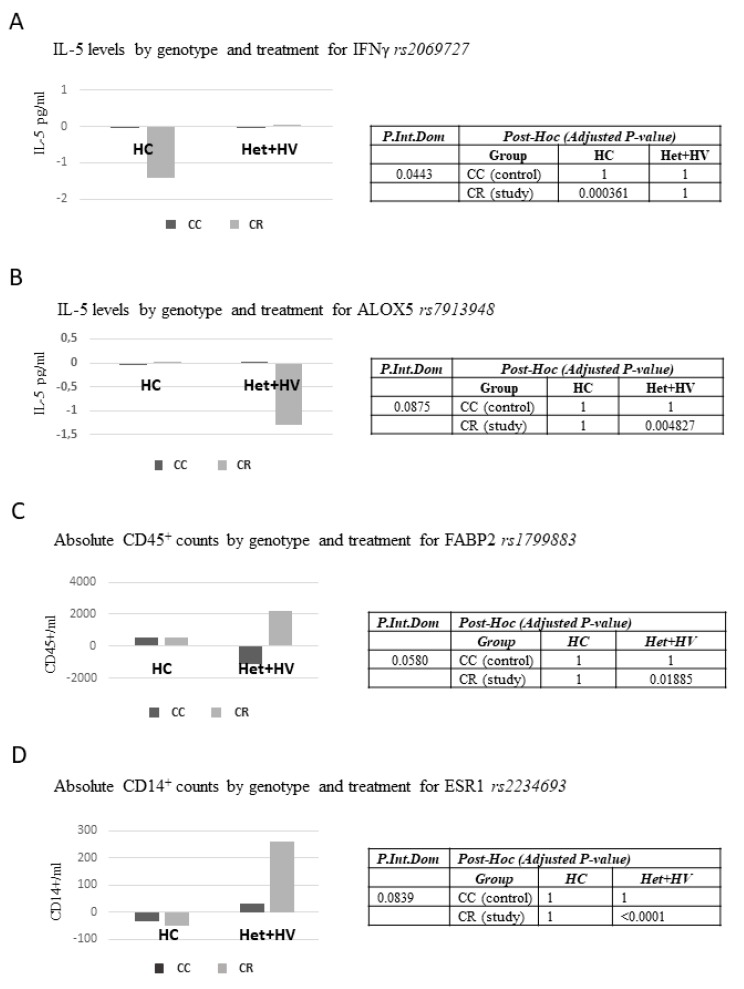
Associations between genetic variants (SNPs) and responses to the nutritional intervention. (**A**) IL-5 levels by genotype and treatment for INFγ *rs*2069727; (**B**) IL-5 levels by genotype and treatment for ALOX5 *rs*7913948, (**C**) Absolute CD45^+^ counts by genotype and treatment for FABP2 *rs*1799883, (**D**) Absolute CD14^+^ counts by genotype and treatment for ESR1 *rs*2234693.

Many studies with formulas to enhance immune system may mask their effects due to the fact of grouping different types of patients. For this purpose, we selected 64 single nucleotide polymorphisms (SNPs) related to inflammation, immune system, obesity, lipid metabolism, redox homeostasis, and cancer to address putative associations between responses to the study variables. All polymorphisms were in Hardy–Weinberg equilibrium except for *rs662799, rs5443,* and *rs6131* SNPs (Supplementary Table S3). The associations between SNP genotypes and changes in the study variables regarding the interaction treatment (CC/CR) x visit were analyzed. Given the small sample size of individuals in the study, the evaluation of the effect of the SNPs genotypes on the evolution of the variables was examined using the dominant model. Figure 5 shows the parameters with a significant interaction (*p* < 0.05), or in the limit of the significance between treatment-gene.

Study group carriers homozygous for the major allele (CC) for *IFNγ rs2069727* SNP displayed significant reduced levels of IL-5 (*p* = 0.000361) (Figure 5A). *IFNγ rs2069727* SNP could be involved in immune responses mediated by macrophage activation. Study group volunteers genotyped AG+AA (carriers of the minor allele) for *ALOX5 rs7913948* SNP displayed significant reduced levels of IL-5 (*p* = 0.004827) (Figure 5B). *ALOX5 rs7913948* is involved in the synthesis of pro-inflammatory and anti-inflammatory molecules. A significant interaction was found between *FABP2 rs1799883* SNP and absolute CD45^+^ counts. The post-hoc analysis indicates that study group carriers of the minor allele (CT, TT) for *FABP2 rs1799883* SNP displayed significant increased numbers in absolute cell counts for CD45^+^ marker (*p* = 0.01885) (Figure 5C). This SNP has been described to be involved in the intracellular transport of long-chain fatty acids, and thus, it may affect cell growth and proliferation of PBMC positive for CD45^+^ marker.

Finally, an interaction close to the limit of significance (*p* = 0.0839) was found between *ESR1 rs2234693* and absolute CD14^+^ counts. The post-hoc analysis revealed that CR group carriers of the minor allele (CT, CC) for *ESR1 rs2234693* SNP displayed significant increased numbers in absolute cell counts for CD14^+^ marker (*p* < 0.00001) (Figure 5D).

## 4. Discussion

This study demonstrates the safety and tolerability of a dietary supplement (CR) based on a supercritical rosemary extract (RE) and alkylglycerols used as bioactive vehicle. These safety features of the dietary supplement open new avenues to its potential use as co-adjutant in different diseases. Rosemary extracts (RE) have been shown to exhibit antitumoral effects, both in vitro and in vivo, in a wide range of cancer types [13,14]. Individual components of RE, such as carnosic acid, carnosol, urosolic acid, and rosmarinic acid, have been described as bioactive agents, although the complete extract displays higher bioactivities due to the synergistic effects of the different compounds. Rosemary extracts synergize with several antitumor agents [14] used in clinics, frequently not only enhancing their effects, but also inhibiting their metabolism and reducing the appearance of resistance [14]. Notably, RE is already approved as an agent for human health by the FDA and the EFSA.

Solubility in the physiological media has been identified as a major drawback for bioactive compounds, which failed to display beneficial effects in clinical studies. Herein, shark liver oil (SLO) enriched in bioactive AKGs (i.e., stimulation of hematopoiesis and immunological defenses and antitumor and anti-metastatic activities [19,20,21,22]) was selected as a bioactive lipid carrier of rosemary extract. AKGs were shown effective for improving bioavailability and effectivity of the extract [23]. The anticancer effects of AKGs have been associated with the recruitment and activation of macrophages as a primary antitumor defense (innate immune system response) and to the increased production of cytokines such as IL-12 and IFN-γ, enhancing the cytotoxic activity of NK cells and cytotoxic T lymphocytes, together with an increase proliferation of pre-activated T cells and NK cells [24]. These effects could be reflected in the significant increase of the monocyte, as well as cytotoxic T cell population (Figure 2). This cellular adaptation supports the ex vivo increased IFN γ production by established PBMC primary cultures from patients receiving the CR (Figure 3).

These effects can synergize with the TLR4 inhibition by carnosic acid [25] from rosemary extract when used as complementary therapeutics. Also, the ‘redox’ control of TLR3 activation by antioxidants from rosemary can modulate an adequate immune system response towards a monocyte immunosurveillance phenotype [26]. The molecular signaling mediated by TLR3 involves STAT3 inducing a Th1 type immune response and a MθM1 polarization of the CD56^−^CD4^+^CD14^+^ population. Although there are controversial results regardless the role of AKG or shark oil containing AKG in the promotion [27] or not [28] of a MθM1 phenotype, our results seem to indicate that the combination of RE with AKG could contribute to a more balanced M1/M2 of the Mθ population [29]. These effects could involve the induction of IFN-γ production from naive and activated T and NK cells, enhancing the cytotoxic activity of NK cells and cytotoxic T lymphocytes (Figure 3) favoring host defense and protection. Similarly, the association of IFN-γ with the CR group of treatment could predispose immune response(s) towards a more tough control of cell growth [30,31]. The observed reduction in the expression of *JAK1* supports the potential of CR to attenuate local inflammation in low grade inflammatory diseases (i.e., metabolic syndrome, obesity, and type 2 diabetes) that could predispose to cancer [32,33].

In addition, *NFE2L2* expression levels significantly diminished in the study group (CR) (*p* = 0.031) compared to control group. Nrf2 is a transcription factor activated by oxidative stress, leading to the expression of antioxidant and detoxifying enzymes and drug transporters (MRPs), as well as anti-apoptotic proteins and proteasomes. Nevertheless, several reports support a role of Nrf2 in oncogenesis. Oncogenes *K-Ras, B-Raf,* and *Myc* mediate the transcription and amplification of Nrf2 in cancer cells, contributing to the oncogenic process by diminishing ROS levels [34]. Thus, the observed downregulation in the study group (CR) suggests an overall improvement towards a reduction of oxidative stress and/or inflammation. Moreover, in patients with cancer, downregulation of Nrf2 is expected to contribute to the reduction of drug resistance and an overall therapy improvement.

*BMP2* expression levels significantly decreased in the study group (CR) (*p* = 0.008) compared to control (CC). BMP2 has recently been described to contribute to the promotion and development of colon cancer stem cells, together with an increase in drug resistance. Thus, the nutritional intervention based of RE-AKG (CR) suggests a positive influence on controlling the pool of colon cancer stem cells (CSC) [35].

*CHKA* expression levels also decreased in the study group (CR), although not significantly (*p* = 0.083). Chokα is a well described oncogene which stimulates proliferation, transformation, invasion, and metastasis, and it also potentiates tumorigenesis of other oncogenes such as RhoA [36]. It is overexpressed in different human tumors, such as breast, lung, prostate, and colon cancer, and it has been proposed a biomarker with prognostic value of the evolution in early stages of non-small-cell lung (NSCL) cancer [37]. Moreover, Chokα activity has been shown to be increased in colon adenocarcinomas relative to normal tissue, and it has been proposed as an important target in cancer therapy [38,39]. Thus, the observed decrease in the study group (CR) compared to control support its benefits in patients with cancer.

Importantly, the observed results in gene expression regulation were also confirmed in SW-620 colon cancer cells treated only with RE, reflecting the specific role of the phytochemicals present in the RE extract.

The association between different genetic variants (single nucleotide polymorphisms, SNPs) and the response to the dietary intervention was analyzed in 64 single nucleotide polymorphisms (SNPs), which have been related to inflammation, immune system, obesity, lipid metabolism, redox homeostasis, and cancer. Given the small sample size to perform the evaluation by genotypes, the results were analyzed by the dominant model.

Study group homozygous carriers for the major allele (CC) of *IFNγ rs2069727* SNP, displayed significant reduced levels of IL-5 (*p* = 0.000361). Study group (CR) volunteers genotyped AG+AA (carriers of the minor allele) for *ALOX5 rs7913948* SNP displayed significant reduced levels of IL-5 (*p* = 0.004827). A significant interaction was also found between *FABP2 rs1799883* SNP and absolute CD45^+^ counts. Study group carriers of the minor allele (CT, TT) for *FABP2 rs1799883* SNP displayed significant increased numbers in absolute cell counts for CD45^+^ marker. To the best of our knowledge, there is a lack of studies in relation to the biological significance of these findings. These results reflect the genetic contribution to the differential responses to this nutritional intervention and warrants further attention and future studies.

One of the main limitations of the study is the age and healthy status of the volunteers, as impaired immune function has been implicated in the declining health and higher incidence of cancer in the elderly [40]. Nevertheless, as shown in this study, the proposed underlying mechanism(s) seems to imply innate immune ‘Toll-like’ receptors (TLRs) driving the polarization of myeloid cells. It has been suggested that TLRs signaling contributes to the pathogenesis of age-related neurodegenerative diseases [41]. Thus, age-related changes compromise the first cellular line of defence, such as neutrophils and macrophages, as well as cytotoxic T lymphocytes activity. In this scenario, the regulatory influence of the developed product increasing a surveillance monocyte population it is not expected to cause ‘deleterious’ health effects. The impaired or improved efficacy of the product will be genetically and physiologically determined by age and physiological status of cancer patients, affecting bone marrow myeloid cell production and immune-senescence. This effect can result similar as that on chemotherapeutic agents, which efficacy or toxicity will be determined by metabolic capacities affected by age. Another limitation of this pilot study is the small sample size, and more studies are needed to confirm the results obtained in this study. These aspects warrant further studies to establish the efficacy of the product in the clinical practice.

In summary, our results support the safety and tolerability of a dietary supplement based on rosemary extract and alkylglycerols as vehicle that is reflected moderate changes in plasmatic leukocyte populations associated to the study intervention.

**Figure 6:**
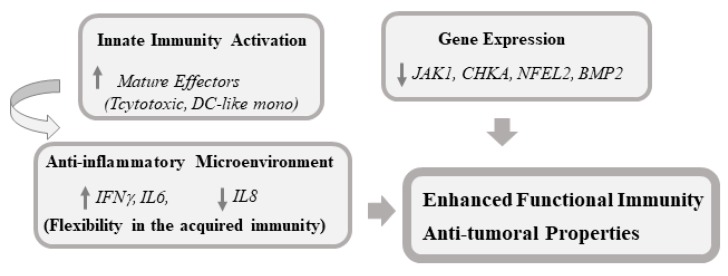
Proposed model of potential main biological benefits of RE-AKG based formula found in this study that encourages its usefulness as co-adjuvant in colon cancer therapy.

The lack of immunosuppressive effects, together with a polarized leukocyte population towards phagocytic and effector cytotoxic T cells in response to CR consumption, supports its inclusion in anticancer strategies. A summary of the potential main biological benefits of the RE-AKG-based formula found in this study that encourages its usefulness as co-adjuvant in colon cancer therapy is presented (Figure 6).

## 5. Conclusions

Herein are investigated the biological and molecular effects of a nutritional supplement based on a *Rosmarinus officinalis* supercritical extract rich in phenolic diterpenes (RE) and shark liver oil rich alkylglycerols (AKG) as potential adjuvant in the therapy of patients suffering from cancer or immune disorders. 60 healthy volunteers participated in a six week, double-blind, randomized parallel pilot study with two study arms: RE-AKG capsules (CR) and control capsules (CC). Safety, tolerability and biological effect of product consumption was confirmed. Our results suggest positive immuno-nutritional effects by mean of (i) activation of innate immune responses, (ii) polarization of this response towards effector phagocytic and cytotoxic natural killer cells (NK) and (iii) an anti-inflammatory cytokine profile which potentially might sustain the immune response towards effector anti-tumor cells. Gene expression modulation supports its potential usefulness in cancer patients. Defined SNPs (Single Nucleotide Polymorphisms) were also associated to differential responses regardless immunological variables. 

## Figures and Tables

**Table 1:** Baseline parameters (V1) (validation of randomization).

	Group CC (*n* = 28)	Group CR (*n* = 30)	*p*
**Age**	28.32 ± 11.39	27.5 ± 9.04	0.963
**Hemogram**			
Leucocytes (10.3/µL)	6.42 ± 1.62	6.33 ± 1.83	0.856
Hemoglobin (g/dL)	13.57 ± 1.2	13.61 ± 1.43	0.896
Eritrocytes (10.6/µL)	4.52 ± 0.35	4.57 ± 0.42	0.669
Platelets (10.3/µL)	231.64 ± 42.35	236.1 ± 47.38	0.846
**Lipidic Profile**			
TC (mg/dL)	174.21 ± 25.90	184.26 ± 29.78	0.319
HDL (mg/dL)	58.64 ± 10.28	61.64 ± 9.57	0.255
LDL (mg/dL)	101 ± 26.02	108.25 ± 27.00	0.302
TG (mg/dL)	72.82 ± 33.20	71.8 ± 31.07	0.904
**Oxidation-inflammation markers**			
CRP_us_ (mg/dL)	0.12 ± 0.15	0.09 ± 0.01	0.308
LDL-ox (U/L)	36.91 ± 11.00	37.09 ± 11.08	0.95
Isoprostanes (ng/mL)	1.06 ± 0.55	1.23 ± 0.91	0.853
Tromboxanes (ng/mL)	983.05 ± 1149.57	1193.59 ± 1662.67	0.783
IFNg (pg/mL)	6.80 ± 8.00	5.27 ± 5.92	0.619
IL 10 (pg/mL)	621.51 ± 1065.24	926.88 ± 1692.32	0.987
IL 1β (pg/mL)	1361.55 ± 833.3	1005.92 ± 825.92	0.05
IL 2 (pg/mL)	0.44 ± 0.24	0.45 ± 0.22	0.73
IL 4 (pg/mL)	2.95 ± 1.55	3.19 ± 1.77	0.363
IL 5 (pg/mL)	0.44 ± 0.22	0.84 ± 1.22	0.844
TNFα (pg/mL)	1940.55 ± 546.88	1671.57 ± 666.93	0.107
**Anthropometric data**			
Weight (kg)	63.11 ± 8.10	65.57 ± 8.31	0.258
BMI (kg/m^2^)	22.4 ± 2.34	22.54 ± 2.19	0.984
Waist (cm)	78.18 ± 7.33	80.96 ± 9.96	0.23
Hip (cm)	98.69 ± 5.01	97.38 ± 8.00	0.456
T Fat Mass (%)	27.36 ± 8.11	25.6 ± 9.52	0.455
T Muscle Mass (%)	32.2 ± 6.05	33.92 ± 7.08	0.497
**Vital constants**			
SBP	116 ± 12.12	118.03 ± 15.35	0.577
DBP	68.79 ± 6.97	69.17 ± 8.91	0.856

Data represents mean ± SD. A *p*-value > 0.05 was considered non-significant. CC (Control capsules), CR (Intervention capsules), TC (Total cholesterol), HDL (High Density Lipoprotein), LDL (Low Density Lipoprotein), TG (Triglycerydes), BMI (Body Mass Index), SBP (Systolic Blood Pressure), DBP (Diastolic Blood Pressure).

**Table 2:** Safety and tolerability of product consumption was assessed by monitoring evolution between visits of (**A**) liver enzymes (glutamyl oxaloacetic transaminase, GOT; glutamic-pyruvatetransaminase, GPT; gamma-glutamyltransferase, GGT; bilirubin; and alkaline phosphatase, AP) and creatinine; (**B**) vital constants (systolic blood pressure, SBP; diastolic blood pressure, DBP; and heart rate, HR); and (**C**) evolution of hematologic profile.

**(A) Liver Enzymes, Total Bilirubin, Creatinine**									
	**CC (Control)**			**CR (Study)**			Effect *		
	**V1**	**V2**	**V3**	**V1**	**V2**	**V3**	**Visit**	**Group**	**Visit × Group**
**GOT (UI/L)**	18.26 (0.73)	18.26 (1.05)	18.07 (0.75)	19.7 (0.86)	18.63 (0.84)	20.57 (0.9)	0.2416	0.1512	0.1512
**GPT (UI/L)**	17.74 (1.4)	16.15 (1.21)	15.7 (1.31)	18.83 (1.47)	16.2 (1.26)	18.63 (1.42)	0.0065	0.368	0.0794
**GGT (UI/L)**	17.99 (2.18)	16.01 (1.5)	15.24 (1.04)	18.63 (1.3)	17.46 (1.14)	18.36 (1.21)	0.0187	0.2867	0.1182
**AP (UI/L)**	57.07 (4.21)	56.19 (3.39)	57.07 (3.66)	54.2 (3.37)	54.83 (2.67)	56.97 (2.89)	0.2637	0.741	0.4248
**TBil (mg/dL)**	0.77 (0.08)	0.7 (0.08)	0.71 (0.08)	0.79 (0.06)	0.7 (0.06)	0.65 (0.05)	0.038	0.9484	0.4705
**Creatinine (mg/dL)**	0.79 (0.02)	-	0.83 (0.02)	0.8 (0.02)	-	0.85 (0.02)	<0.001	0.5661	0.5346
**(B) Vital Constants**									
	**CC (Control)**			**CR (Study)**			Effect *		
	**V1**		**V3**	**V1**		**V3**	**Visit**	**Group**	**Visit × Group**
**SBP (mmHg)**	115.85 (2.28)		110.69 (2.31)	118.03 (2.8)		115.37 (2.63)	0.0091	0.1588	0.3909
**DBP (mmHg)**	69 (1.21)		69.88 (1.7)	69.17 (1.65)		69.28 (1.64)	0.6083	0.9966	0.6844
**HR (beat/min)**	71.38 (2.46)		70.08 (2.34)	67.21 (2.59)		66.1 (1.94)	0.3559	0.1254	0.9132
**(C) Hematological Profile**									
	**CC (Control)**			**CR (Study)**			Effect *		
	**V1**		**V3**	**V1**		**V3**	**Visit**	**Group**	**Visit × Group**
**Erythrocytes (10.6/µL)**	4.53 (0.07)		4.42 (0.07)	4.57(0.08)		4.46 (0.08)	<0.001	0.5753	0.8106
**Erythrocytes (%)**	40.84 (0.57)		39.73 (0.58)	40.95 (0.7)		39.94 (0.67)	<0.001	0.5927	0.8402
**Hemoglobin (g/dL)**	13.63 (0.23)		13.47 (0.22)	13.61 (0.26)		13.52 (0.25)	0.0792	0.7324	0.6625
**Leukocytes (10.3/µL)**	6.4 (0.32)		6.01 (0.29)	6.33 (0.33)		6.5 (0.32)	0.6178	0.6266	0.1667
**Lymphocytes (10.3/µL)**	2.15 (0.09)		2.14 (0.1)	2.2 (0.09)		2.3 (0.14)	0.408	0.4449	0.3247
**Neutrophils (10.3/µL)**	3.53 (0.28)		3.2 (0.22)	3.41 (0.28)		3.47 (0.23)	0.4645	0.8303	0.2574
**Monocytes (10.3/µL)**	0.53 (0.03)		0.5 (0.03)	0.51 (0.02)		0.5 (0.02)	0.2564	0.7444	0.4594
**Basophils (10.3/µL)**	0.06 (0)		0.05 (0)	0.07 (0)		0.06 (0)	<0.001	0.0962	0.9151
**Platelets (10.3/µL)**	231.33 (8.3)		217.59 (7.78)	236.1 (8.65)		228.67 (8.26)	0.0048	0.4715	0.3886

* Effect: Visit indicates evolution in the same group from V1 to V3; group indicates the differences between groups (CC: control capsules), CR: intervention capsules) regardless of time; and visit × group indicates the differences in evolution (V1 to V3) between groups as a result of the treatment (CC, CR). The *p*-value of the interaction was adjusted by sex, age, and BMI. Data expressed as mean (SEM). A *p*-value > 0.05 was considered non-significant.

**Table 3:** Evolution between visits of (**A**) inflammation and oxidation parameters (LDLox, total isoprotanes, tromboxanes TBX2, and C-reactive protein); (**B**) lipid profile (total cholesterol, TC; high density lipoprotein, HDL; triglycerides, TG; and low density lipoprotein, LDL); and (**C**) anthropometric data (weight; body mass index, BMI; fat mass, FM; muscle mass, MS; and waist circumference, Wc).

**(A) Markers of Inflammation and Oxidation**							
	**CC (Control)**		**CR (Study)**		Effect *		
	**V1**	**V3**	**V1**	**V3**	**Visit**	**Group**	**Visit × Group**
**LDLox (U/L)**	36.91 (2.12)	34.15 (1.81)	37.09 (2.02)	38.66 (2.14)	0.5902	0.3653	0.0196
**T isop (ng/mg creat)**	0.87 (0.08)	0.84 (0.06)	0.8 (0.09)	0.95 (0.07)	0.3129	0.8948	0.1045
**TXB2 (ng/mg creat)**	713.1 (128.16)	709.39 (120.37)	713.74 (178.88)	879.44 (187.51)	0.5506	0.7363	0.4338
**CRP (mg/dL)**	0.11 (0.03)	0.09 (0.02)	0.09 (0.02)	0.07 (0.01)	0.0826	0.1725	0.7098
**(B) Lipid Profile**							
	**CC (Control)**		**CR (Study)**		Effect *		
	**V1**	**V3**	**V1**	**V3**	**Visit**	**Group**	**Visit × Group**
**TC (mg/dL)**	174.96 (5.02)	171.05 (5.02)	184.26 (5.44)	185.77 (5.93)	0.6062	0.0723	0.1745
**HDL (mg/dL)**	58.41 (2)	65.67 (2.65)	61.64 (1.75)	65.59 (2.7)	<0.001	0.6235	0.1491
**TG (mg/dL)**	73.19 (6.5)	70.74 (5.9)	71.8 (5.67)	75.2 (7.73)	0.8527	0.8138	0.4571
**LDL (mg/dL)**	101.91 (5.02)	91.23 (5.29)	108.25 (4.93)	105.13 (5.6)	<0.001	0.1078	0.0434
**(C) Anthropometric Data**							
	**CC (Control)**		**CR (Study)**		Effect *		
	**V1**	**V3**	**V1**	**V3**	**Visit**	**Group**	**Visit × Group**
**Weight (kg)**	63.11 (1.59)	62.57 (1.58)	65.57 (1.52)	65.35 (1.58)	0.0222	0.0276	0.3273
**BMI (kg/m2)**	22.3 (0.45)	22.11 (0.42)	22.54 (0.4)	22.46 (0.39)	0.0031	<0.001	0.3137
**FM (%)**	26.95 (1.57)	26.02 (1.56)	25.6 (1.74)	25.5 (1.71)	0.0125	0.228	0.0523
**MM (%)**	32.42 (1.19)	32.92 (1.2)	33.92 (1.29)	33.92 (1.3)	0.0727	0.0761	0.0787
**Wc(cm)**	78.11 (1.43)	77.75 (1.37)	80.96 (1.82)	79.14 (1.19)	0.2267	0.0954	0.4164

* Effect: Visit indicates evolution in the same group from V1 to V3; group indicates the differences between groups (CC, CR) regardless of time; and visit × group indicates the differences in evolution (V1 to V3) between groups as a result of the treatment (CC, CR). The *p*-value of the interaction was adjusted by sex, age, and BMI. Data expressed as mean (SEM). A *p*-value > 0.05 was considered non-significant.

**Table 4:** Evolution in absolute counts of cluster differentiation (CD) markers.

Cluster Differentiation Markers Counts							
	CC (Control)		CR (Study)		Effect *		
	V1	V3	V1	V3	Visit	Group	Visit × Group
**CD45^+^ (cs/mL)**	6458.99 (457.29)	6231.24 (474.55)	6260.18 (403.99)	7764.86 (378.69)	0.0491	0.2578	0.0207
**CD3^+^ (cs/mL)∆**	1615.72 (124.1)	1744.94 (140.34)	1581.5 (93.34)	2012.5 (161.31)	0.0018	0.5577	0.0678
**CD4^+^ (cs/mL)∆**	960.67 (66.18)	1045.5 (77.89)	902.11 (59.85)	1149.33 (89.98)	<0.001	0.9008	0.0714
**CD8^+^ (cs/mL) ∆**	526.94 (68.13)	555.56 (63.82)	524.28 (36.67)	667.11 (62.62)	0.0047	0.5025	0.0563
**CD14^+^ (cs/mL)**	473.06 (39.68)	471.22 (40.84)	409.94 (28.53)	565.72 (42.81)	0.0144	0.7858	0.0126
**CD16^+^ (cs/mL)**	3485.56 (362.15)	3183.78 (310.67)	3253.83 (274.68)	3935.72 (224.94)	0.365	0.3393	0.0318
**CD56^+^ (cs/mL)**	429.61 (51.53)	407.78 (46.1)	415.33 (40.05)	516.11 (50.95)	0.2417	0.4904	0.0716

* Effect: Visit indicates the evolution in the same group from the beginning to the end of the intervention; group indicates the differences between groups regardless of time; and visit × group indicates the differences in evolution between groups as a result of the treatment received (CC or CR). The *p*-value of the interaction was adjusted by sex, age, and BMI. Data expressed as mean (SEM). A *p*-value > 0.05 was considered non-significant. Analysis performed on a subsample (*n* = 18) for group CC and (*n* = 18) for group CR. ∆ Counts on CD45^+.^

## Supplementary Materials

The following are available online at https://www.mdpi.com/2072-6643/11/9/2001/s1, Table S1. Control Capsules (CC) and Study Capsules (CR) composition; Table S2. Table of the selected genes and pathways analyzed in the study; Table S3. Table of the selected SNPs with frequencies and Hardy–Weinberg equilibrium (HWE) analysis; Figure S1. Principal Components Analysis (PCA) plots of the evolution (V1 and V3) of the ex vivo cytokines produced by LPS stimulated PBMCs for CR and CC. Positively identified cytokines with major influence in PCA were IFNγ, and IL-6 and IL8, with a major weight of PC1 (Eigenvalue, 1.21) versus the PC2 (Eigenvalue, 0.79) represented by other cytokines.

## Abbreviations

AKG	Alkylglycerols
AP	Alkaline phosphatase
APC	Antigen-presenting cell
Bil	Bilirubin
BMI	Body mass index
*BMP2*	Bone morphogenetic protein 2
*CHKA*	Choline kinase A
CRP	C reactive protein
CC	Control capsules
CR	Intervention capsules
DC	Dendritic cell
GGT	Gamma glutamyltransferase
GOT	Glutamyl oxaloacetic transaminase
GPT	Glutamic-pyruvatetransaminase
HDL	High-density lipoprotein
IFN	Interferon
IL	Interleukin
*JAK1*	Janus kinase 1
LDL	Low-density lipoprotein
LPS	Lipopolysaccharide
Mθ	Macrophages
MC	Monocytes
*NFE2L2*	Nuclear factor (erythroid-derived 2)-like 2
PBMC	Peripheral blood mononuclear cells
PCA	Principal component analysis
RE	Rosmarinus officinalis L. extract
