# Supplementary material for: Tolerability and Safety of a Nutritional Supplement with Potential as Adjuvant in Colorectal Cancer Therapy: A Randomized Trial in Healthy Volunteers
Source: Nutrients. 2019 Aug 24;11(9):2001. doi: 10.3390/nu11092001 (PMC6769991; doi:10.3390/nu11092001)
Supplement: Supplementary file 1 [file nutrients-11-02001-s001.pdf]

Supplementary Table S1. Control Capsules (CC) and Study Capsules (CR) composition

**CC Capsules Composition**	**mg**	**Bioactive compound**
Sunflower oil	1.100	
Gelatin	318,019	
Glycerin	118,750	
Clorophill E-140	1,923	
CAPSULE TOTAL WEIGHT	1.538,692	

**CR Capsules Composition**	**mg**	**Bioactive compound**
Shark liver oil 20% alkylglycerols (Gustav Heess)	750	***150 mg alkylglycerols***
Rosemary Antioxidant extract, 25 % Diterpene Phenols, Type Nº 027.020 (*Rosmarinus officinalis L.)*	45	***11,25 mg Diterpene Phenols***
Glyceril-monoestearate	30	
Sunflower oil	263	
Soy lecithin VEROLEC 56	12	
Gelatin	318,019	
Glycerin	118,750	
Clorophill E-140	1,923	
CAPSULE TOTAL WEIGHT	1.538,692	

Supplementary Table S2. Table of the selected genes and pathways analyzed in the study

Pathway	Gen	Gene name
**Inflammation, Immunomodulation**	*IL1B*	Interleukin 1, Beta
	TNF (TNFA)	Tumor Necrosis Factor
	MAPK1	Mitogen-Activated Protein Kinase 1
	PTK2B	Protein Tyrosine Kinase 2 Beta
	STAT3	Signal Transducer Activator Of Transcription 3
	JAK1	Janus Kinase 1
	JAK3	Janus Kinase 3
	NFKB	Nuclear Factor Of Kappa Light Polypeptide Gene Enhancer In B-Cells 1
	NLRP3	NLR Family, Pyrin Domain Containing 3
	CCL2 (MCP-1)	Chemokine (C-C Motif) Ligand 2
	CXCR1	Chemokine (C-X-C Motif) Receptor 1
	CSF2	Colony Stimulating Factor 2 (Granulocyte-Macrophage)
	CCL5(RANTES)	Chemokine (C-C Motif) Ligand 5
	CCR5	Chemokine (C-C Motif) Receptor 5 (Gene/Pseudogene)
	PLCG1	Phospholipase C, Gamma 1
	PRKCD	Protein Kinase C, Delta
	ADIPOQ	Adiponectin, C1Q And Collagen Domain Containing
	BMP2	Bone Morphogenetic Protein 2
	LIF	Leukemia Inhibitory Factor
	TGFB2	Transforming Growth Factor, Beta 2
	IFNG	Interferon, Gamma
	IL2	Interleukin 2
	IL6	Interleukin 6
	TLR4	Toll-Like Receptor 4
**Response to Oxidative Stress**	*NOS2*	Nitric Oxide Synthase 2, Inducible
	NOX5	NADPH Oxidase, EF-Hand Calcium Binding Domain 5
	NCF2	Neutrophil Cytosolic Factor 2
	SOD1	Superoxide Dismutase 1, Soluble
	GPX1	Glutathione Peroxidase 1
	PRDX5	Peroxiredoxin 5
	NFE2L2	Nuclear Factor, Erythroid 2-Like 2
**RE Molecular Targets in CRC**	*DTYMK*	Deoxythymidylate Kinase (Thymidylate Kinase)
	TK1	Thymidine Kinase 1, Soluble
	GCNT3	Glucosaminyl (N-Acetyl) Transferase 3, Mucin Type
	PLA2G7	Phospholipase A2, Group VII (Platelet-Activating Factor Acetylhydrolase, Plasma)
**Lipid Metabolism, Adipogenesis, Obesity**	*PPARA*	Peroxisome Proliferator-Activated Receptor Alpha
	PPARG	Peroxisome Proliferator-Activated Receptor Gamma
	CEBPA	CCAAT/Enhancer Binding Protein (C/EBP), Alpha
	CEBPB	CAAT/Enhancer Binding Protein (C/EBP), Beta
	SREBF1	Sterol Regulatory Element Binding Transcription Factor 1
	FASN	Fatty Acid Synthase
	IRS2	Insulin Receptor Substrate 2
	ACSL4	Acyl-CoA Synthetase Long-Chain Family Member 4
	GPD2	Glycerol-3-Phosphate Dehydrogenase 2 (Mitochondrial)
	CHKA	Choline Kinase Alpha
	LEP	Leptin
	LEPR	Leptin Receptor

Supplementary Table S3. Table of the selected SNPs with frequencies and Hardy Weinberg equilibrium (HWE) analysis

Gene Symbol	Gene Name	SNP	Major allele Homozygote			Heterozygote			Minor allele Homozygote			Allele1 (MAF)	Allele 2	HWE
				n	%		n	%		n	%			*P*
ACE	Angiotensin I converting enzyme	rs4343	GG	24	41,38	AG	25	43,1	AA	9	15,52	0,3707	0,6293	0,7108
ADIPOQ	Adiponectin	rs2241766	TT	33	56,9	GT	23	39,66	GG	2	3,45	0,2328	0,7672	0,5857
ADIPOQ	Adiponectin	rs1501299	GG	32	55,17	GT	25	43,1	TT	1	1,72	0,2328	0,7672	0,2019
ADRB1	Adrenoceptor beta 1	rs1801253	CC	37	63,79	CG	17	29,31	GG	4	6,9	0,2155	0,7845	0,4853
ADRB3	Adrenoceptor beta 3	rs4994	AA	51	87,93	AG	7	12,07				0,0603	0,9397	0,5284
AGT	Angiotensinogen	rs699	AA	16	27,59	AG	32	55,17	GG	10	17,24	0,4483	0,5517	0,4951
AGTR1	Angiotensin II receptor, type 1	rs5186	AA	34	58,62	AC	19	32,76	CC	5	8,62	0,25	0,75	0,4942
ALOX5	Arachidonate 5-lipoxygenase	rs7913948	GG	40	68,97	AG	15	25,86	AA	3	5,17	0,181	0,819	0,5453
APO B	Apoliprotein B	rs693	GG	17	29,31	AG	25	43,1	AA	16	27,59	0,4914	0,5086	0,3931
APO B	Apoliprotein B	rs1367117	GG	32	56,14	AG	18	31,58	AA	7	12,28	0,2807	0,7193	0,2807
APOA2	Apoliprotein A2	rs5082	AA	49	84,48	AG	9	15,52				0,0776	0,9224	0,7908
APOA5	Apoliprotein A5	rs662799	AA	51	87,93	AG	5	8,62	GG	2	3,45	0,0776	0,9224	0,027
APOE	Apoliprotein E	rs7412	CC	55	94,83	CT	3	5,17				0,0259	0,9741	1
APOE	Apoliprotein E	rs429358	TT	45	77,59	CT	13	22,41				0,1121	0,8879	0,6965
BDNF	Brain-derived neurotrophic factor	rs6265	CC	36	62,07	CT	18	31,03	TT	4	6,9	0,2241	0,7759	0,6057
CETP	Cholesteryl ester transfer protein, plasma	rs708272	GG	25	43,1	AG	27	46,55	AA	6	10,34	0,3362	0,6638	0,9023
CLOCK	Clock circadian regulator	rs1801260	AA	29	50	AG	26	44,83	GG	3	5,17	0,2759	0,7241	0,5021
CLOCK	Clock circadian regulator	rs3749474	CC	24	42,86	CT	29	51,79	TT	3	5,36	0,3125	0,6875	0,1958
CLOCK	Clock circadian regulator	rs4580704	CC	20	34,48	CG	31	53,45	GG	7	12,07	0,3879	0,6121	0,4543
COMT	Catechol-O-methyltransferase	rs4680	GG	22	37,93	AG	26	44,83	AA	10	17,24	0,3966	0,6034	0,7778
CRP	C-reactive protein	rs1130864	GG	28	48,28	AG	25	43,1	AA	5	8,62	0,3017	0,6983	0,9164
CSF1	Colony stimulating factor 1	rs333970	AA	20	34,48	AC	31	53,45	CC	7	12,07	0,3879	0,6121	0,4543
CYP2D6	Cytochrome P450, family 2, subfamily D, polypeptide 6	rs28371725	CC	48	84,21	CT	9	15,79				0,0789	0,9211	0,7991
ESR1	Estrogen receptor 1	rs2234693	TT	23	39,66	CT	24	41,38	CC	11	18,97	0,3966	0,6034	0,4092
ESR1	Estrogen receptor 1	rs9340799	AA	25	43,1	AG	27	46,55	GG	6	10,34	0,3362	0,6638	0,9023
FABP2	Fatty acid binding protein 2	rs1799883	CC	27	46,55	CT	29	50	TT	2	3,45	0,2845	0,7155	0,1381
FADS1	Fatty acid desaturase 1	rs174546	CC	28	48,28	CT	29	50	TT	1	1,72	0,2672	0,7328	0,0659
FASLG	Fas ligand (TNF superfamily member 6)	rs763110	CC	22	37,93	CT	26	44,83	TT	10	17,24	0,3966	0,6034	0,7778
FTO	Fat mass and obesity associated	rs1421085	TT	21	36,21	CT	26	44,83	CC	11	18,97	0,4138	0,5862	0,7042
FTO	Fat mass and obesity associated	rs9939609	TT	22	37,93	AT	29	50	AA	7	12,07	0,3707	0,6293	0,7357
GHRL	Ghrelin/obestatin prepropeptide	rs696217	GG	48	84,21	GT	9	15,79				0,0789	0,9211	0,7991
GNB3	Guanine nucleotide binding protein, beta polypeptide 3	rs5443	CC	21	36,84	CT	34	59,65	TT	2	3,51	0,3333	0,6667	0,0157
IFI30	Interferon, gamma-inducible protein 30	rs11554159	GG	32	55,17	AG	23	39,66	AA	3	5,17	0,25	0,75	0,861

IFNA5	Interferon, alpha 5	rs10757212	GG	39	67,24	AG	14	24,14	AA	5	8,62	0,2069	0,7931	0,0925
IFNG	Interferon, gamma	rs2069727	CC	17	29,31	CT	29	50	TT	12	20,69	0,4569	0,5431	0,8756
IL10	Interleudin, 10	rs1800896	TT	21	36,84	TC	30	52,63	CC	6	10,53	0,3684	0,6316	0,4392
IL1B	Interleukin 1, beta	rs1143634	GG	36	62,07	GA	18	31,03	AA	4	6,9	0,2241	0,7759	0,6057
IL1B	Interleukin 1, beta	rs1143643	CC	24	41,38	CT	30	51,72	TT	4	6,9	0,3276	0,6724	0,2731
IL2	Interleukin 2	rs2069762	AA	26	45,61	CA	26	45,61	CC	5	8,77	0,3158	0,6842	0,8457
IL6	Interleukin 6	rs1800797	GG	20	34,48	AG	30	51,72	AA	8	13,79	0,3966	0,6034	0,6804
IL6	Interleukin 6	rs1800795	GG	21	36,21	CG	29	50	CC	8	13,79	0,3879	0,6121	0,8383
LEP	Leptin	rs12535708	CC	20	34,48	AC	31	53,45	AA	7	12,07	0,3879	0,6121	0,4543
LEPR	Leptin receptor	rs1137101	AA	17	29,31	AG	31	53,45	GG	10	17,24	0,4379	0,5621	0,6529
LEPR	Leptin receptor	rs1137100	AA	32	56,14	AG	23	40,35	GG	2	3,51	0,2368	0,7632	0,5593
LPL	Lipoprotein lipase	rs328	CC	38	65,52	CG	20	34,48				0,1724	0,8276	0,2314
MC4R	Melanocortin 4 receptor	rs17782313	TT	44	75,86	CT	13	22,41	CC	1	1,72	0,1293	0,8707	0,6189
MTHFR	Methylenetetrahydrofolate reductase	rs1801133	GG	19	32,76	GA	31	53,45	AA	8	13,79	0,4052	0,5948	0,5314
NOS3	Nitric oxide synthase 2, inducible	rs1799983	GG	25	43,1	GT	24	41,38	TT	9	15,52	0,3621	0,6379	0,5621
NPY	Neuropeptide Y	rs16139	TT	46	88,46	CT	6	11,54				0,0577	0,9423	0,4296
NR3C1	Nuclear receptor subf 3, C, member 1 (glucocorticoid receptor)	rs6196	AA	36	63,16	AG	20	35,09	GG	1	1,75	0,193	0,807	0,5459
ORL1	Oxidized low density lipoprotein receptor 1	rs3736235	TT	16	27,59	TC	29	50	CC	13	22,41	0,4741	0,5259	0,85
PLIN1	Perilipin 1	rs894160	CC	30	52,63	CT	21	36,84	TT	6	10,53	0,2895	0,7105	0,5905
PON1	Paraoxonase 1	rs662	TT	32	55,17	CT	20	34,48	CC	6	10,34	0,2759	0,7241	0,4332
PPARA	Peroxisome proliferator-activated receptor alpha	rs6008259	GG	38	65,52	AG	19	32,76	AA	1	1,72	0,181	0,819	0,6653
PPARγ	Peroxisome proliferator-activated receptor gamma	rs1801282	CC	53	91,38	CG	4	6,9	GG	1	1,72	0,0517	0,9483	0,3132
PTGS2	Prostaglandin-endoperoxide synthase 2	rs2066826	CC	33	56,9	CT	24	41,38	TT	1	1,72	0,2241	0,7759	0,2557
SELP	Selectin P	rs6131	CC	19	39,58	CT	29	60,42				0,3021	0,6979	0,0041
SIRT1	Sirtuin 1	rs7069102	GG	11	28,21	CG	24	61,54	CC	4	10,26	0,4103	0,5897	0,1475
SREBF1	Sterol regulatory element binding transcription factor 1	rs11868035	GG	29	50	AG	22	37,93	AA	7	12,07	0,3103	0,6897	0,5281
TCF7L2	Transcription factor 7-like 2 (T-cell specific,	rs7903146	CC	24	42,11	CT	21	36,84	TT	12	21,05	0,3947	0,6053	0,1287
TNFRSF1A	Tumor necrosis factor receptor superfamily, member 1A	rs767455	TT	21	36,21	CT	29	50	CC	8	13,79	0,3879	0,6121	0,8383
TNFα	Tumor necrosis factor	rs1800629	GG	48	82,76	AG	10	17,24				0,0862	0,9138	0,8732
UCP2	Uncoupling protein 2	rs659366	CC	21	36,21	CT	31	53,45	TT	6	10,34	0,3707	0,6293	0,3704
UCP3	Uncoupling protein 3	rs1800849	GG	39	67,24	AG	17	29,31	AA	2	3,45	0,181	0,819	0,7592

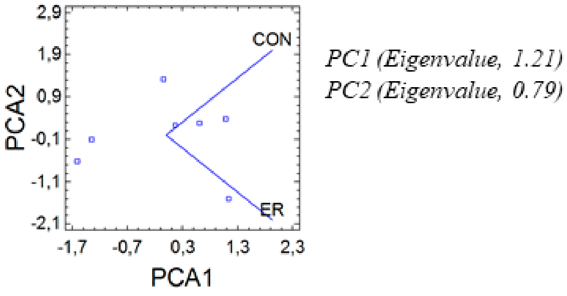

Figure S1. Principal Components Analysis (PCA) plots of the evolution (V1 and V3) of the *ex vivo* cytokines produced by LPS stimulated PBMCs for CR and CC. Positively identified cytokines with major influence in PCA were IFNγ, and IL-6 and IL8, with a major weight of PC1 (Eigenvalue, 1.21) *versus* the PC2 (Eigenvalue, 0.79) represented by other cytokines.
